# Building Localized NADP(H) Recycling Circuits to Advance Enzyme Cascadetronics

**DOI:** 10.1002/anie.202414176

**Published:** 2025-02-11

**Authors:** Ryan A. Herold, Christopher J. Schofield, Fraser A. Armstrong

**Affiliations:** ^1^ Department of Chemistry University of Oxford Mansfield Road Oxford OX1 3QY United Kingdom; ^2^ Chemistry Research Laboratory Department of Chemistry and the Ineos Oxford Institute for Antimicrobial Research University of Oxford Mansfield Road Oxford OX1 3QY United Kingdom; ^3^ Current Address: Department of Chemistry and Biochemistry University of California San Diego La Jolla, CA 92093 USA

**Keywords:** Biocatalysis, Enzyme Cascade, Hydrogen Borrowing, Nanoconfinement, Cofactor Recycling

## Abstract

The catalytic action of enzymes in a cascade trapped within a mesoporous electrode material is simultaneously energized, controlled and observed through the efficient, reversible electrochemical NAD(P)(H) recycling catalyzed by one of the enzymes. In their nanoconfined state, nicotinamide cofactors are tightly channeled current carriers, mediating multi‐step reactions in either direction (oxidation or reduction) with a rapid response time. By incorporating a hydrogen‐borrowing enzyme pair, the internal action of which opposes the external voltage *bias* driving oxidation or reduction, a reduction process can be performed under overall oxidizing conditions, and vice versa. The power of the method to control and resolve complex metabolic pathways is demonstrated using a non‐linear, three‐enzyme cascade extended by incorporating a fourth enzyme, urease. The latter generates in situ ammonia, which is enzymatically consumed in a *reductive* process, but the immediate current response to each addition of urea is observed, unusually, by applying an *oxidizing* potential. A practical consequence is that enzyme‐catalyzed electrochemical reduction reactions requiring anaerobic conditions (to avoid O_2_ interference) can readily be studied under ambient aerobic conditions. The results illustrate how a complex enzyme cascade confined within a porous electrode and connected to an electrical power source manifests characteristics associated with electronic circuits.

## Graphical abstract

A consequence of the high catalytic activities and selectivities of many enzymes is that cascades enclosed within a mesoporous conducting material using NADP(H) and intermediates as internal current carriers display the almost instantaneous information‐transfer properties usually associated with electronic circuits. Such collective behavior of enzymes is demonstrated by incorporating a hydrogen‐borrowing pair to compete against the external potential bias.

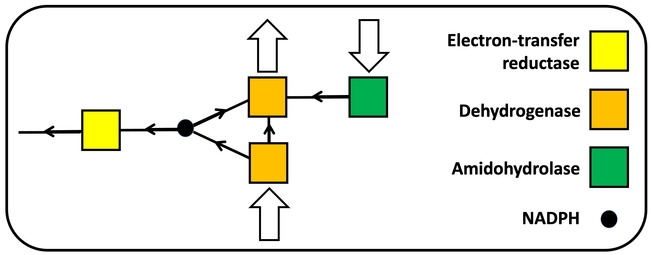

## Introduction

The Electrochemical Leaf (e‐Leaf) is a concept and platform for engaging and operating nanoconfined enzyme cascades in a porous inorganic electrode material.[^1^, ^2^] As well as artificially reproducing the physical conditions of high local concentration and crowding that enzymes experience in living cells,[^3^, ^4^, ^5^] the e‐Leaf allows direct control and observation of complex electrochemically‐driven catalytic processes.

The key catalytic components are: (i) a transducing enzyme (E1), typically photosynthetic ferredoxin NADP^+^‐reductase (FNR), which undergoes direct electron exchange with the ITO material and thereby catalyzes the reversible electrocatalytic recycling of NAD(P)(H);^[6]^ (ii) a second enzyme (E2) that can be any of the myriad dehydrogenases using NAD(P)(H);^[7]^ and (iii) the ubiquitous hydride‐transferring NAD(P)(H) cofactor itself, which is effectively localized in the porous network.^[3]^ Further enzymes of many classes (not necessarily dehydrogenases) can be introduced to create bespoke cascades.[^8^, ^9^] The e‐Leaf is named because the rapid catalytic cycling of NADPH by FNR is a central step in photosynthesis, distributing reducing power for CO_2_ assimilation and other processes.^[10]^ As the most efficient NADP(H) recycling catalyst yet discovered, FNR operates reversibly, the electrode potential imposing exquisite control of catalytic activity in each direction.[^6^, ^11^] The mesoporous electrode material consists of metallic oxide nanoparticles (typically indium tin oxide, ITO) electrophoretically deposited to a depth of 3–6 mm on a suitable conductive support, such as pyrolytic graphite, ITO glass or titanium foil.[^7^, ^12^] Dissolved enzymes applied to the surface spontaneously enter the pores, leading to local concentrations approaching the millimolar range.[^1^, ^3^] There is increasing interest in finding new ways to manipulate complex enzyme reaction networks, both natural and synthetic, for catalysis, sensing and advancing basic fundamental insight.[^13^, ^14^, ^15^, ^16^] With the e‐Leaf, such networks are not only assembled easily, but they are also rendered highly interactive – in stark contrast with non‐electrochemical approaches to cascade scaffolding in which reactions are initiated and driven by chemicals or light, and rates are determined by monitoring how the concentration of a species (such as NADH) changes with time.[^17^, ^18^, ^19^, ^20^]

The e‐Leaf has been applied to a range of topics in biocatalysis, achieving levels of control well above that of (or not possible in) solution‐based systems. Examples include extended cascades,^[9]^ synthesis,^[12]^ coupling with kinases,^[8]^ deracemization of secondary alcohols,^[21]^ and a detailed investigation of the kinetic mechanism of a cancer drug.^[22]^ The scope is extensive because a very broad selection of components is available (natural enzymes and natural/unnatural variants). Since FNR is a highly selective and reversible electrocatalyst which becomes concentrated within the metal oxide pores, free or polymer‐based electron mediators,[^23^, ^24^] the mainstay of traditional bioelectrochemistry, are not required.

The crowding and nanoconfinement that the enzymes experience, combined with their typically high catalytic specificities (reflected in *k*_cat_/*K*_M_) and substrate selectivities, help ensure that cascade intermediates have a high probability of being processed within the porous electrode network before they can escape to solution.^[2]^ Nanoconfinement of NADP(H) greatly increases its efficiency as an exchangeable cofactor; indeed, a turnover number of >150,000 was achieved using only NADP(H) introduced to the electrode as “cargo” on a dehydrogenase.^[3]^

Within the nanoconfined enzyme network, nicotinamide cofactors become discrete electrical current carriers which, like electrons, mediate the rapid flow of energy and information, opening the way to handling myriad enzymes as if they were electronic circuit components (“cascadetronics”).^[2]^ As with a circuit board, enzymes can be slotted in as *internal* components, to be tested, investigated and exploited – their characteristics displayed through their response to the electrode potential (the *external bias*, usually a DC voltage that controls and powers the system). Channeling of the current extends across each reaction step upstream of NAD(P)(H) recycling wherever an intermediate is retained in the pores: in this case, all the enzymes become effectively electroactive – regardless of whether or not they catalyze an electron‐transfer reaction.[^8^, ^9^]

We now describe the significant effect of incorporating a hydrogen‐borrowing enzyme pair into an e‐Leaf cascade. Adding the internal enzyme circuit modulates the NADP(H) current flow, enabling a new capability – that of driving reduction reactions under overall oxidizing (aerobic) conditions. As with the reverse situation (i.e. driving an oxidizing reaction under reducing conditions), this situation occurs in multiple metabolic processes in living cells, but the effects of modulating factors on enzyme cascades are difficult to study using conventional approaches and models. Nicotinamide cofactors are selective and inert to O_2_, contrasting with promiscuous electron mediators for which any such organization of electron flow would be difficult. The concept is demonstrated by detecting the real‐time activity of the ammonia‐producing enzyme urease via reductive amination, measured at an oxidizing electrode.

## Results and Discussion

### The Concept

**Figure 1:**
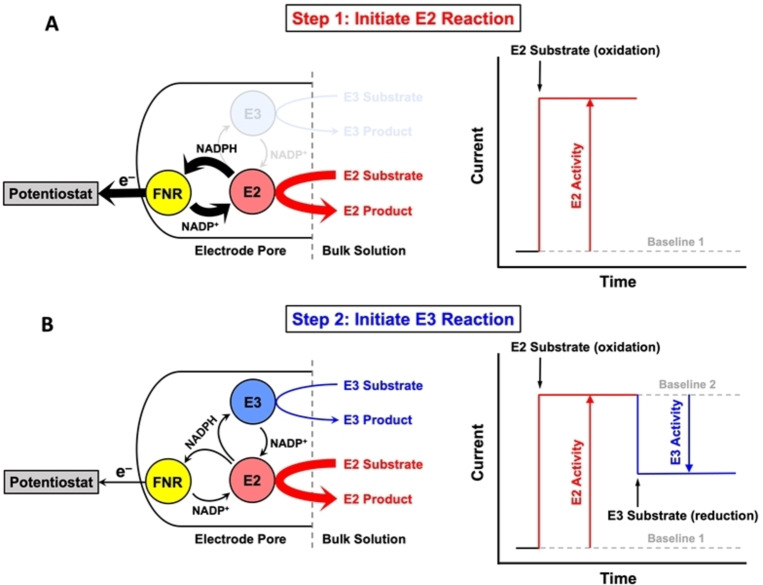
Extension of the e‐Leaf concept: nicotinamide cofactors as nanoconfined current carriers and the effect of introducing a competing NADP(H) current source. (A) Step 1: 3 enzymes (FNR (E1), and two dehydrogenases E2 and E3) are co‐loaded into a mesoporous ITO electrode. When the NADP^+^‐dependent E2 oxidation reaction is initiated, an oxidation current proportional to the E2 turnover rate is measured by direct electron transfer from FNR to the electrode, because NADP(H) is rapidly recycled in the electrode between FNR and E2. (B) Step 2: the NADPH‐dependent E3 reduction reaction is initiated, whereby E3 now competes with FNR for the NADPH produced by E2, creating an alternative competing pathway for NADP^+^ recycling and producing a decrease in oxidation current that is proportional to E3 activity. Changes in the relative magnitude*s* of the two competing pathways are represented by the arrow thickness.

e‐Leaf cascades are conveniently represented as maps depicting the various enzymes confined in an electrode pore and emphasizing their connectivities in terms of adjacent positions in the reaction sequence – physical proximity being considered to have limited relevance under these conditions.^[25]^ Figure 1 shows a generic three‐enzyme cascade designed to enable an enzyme‐catalyzed *reduction* reaction to be simultaneously energized and monitored electrochemically under oxidizing conditions. The arrow showing electrons being removed from the cascade signifies a positive (oxidizing) bias. Aside from FNR (E1), which catalyzes reversible electrochemical NADP^+^/NADPH interconversion[^6^, ^26^] (formal potential, −0.35 V at pH 8), E2 is an NADP^+^‐dependent enzyme catalyzing an oxidation, and E3 is another NADPH‐dependent enzyme that catalyzes a reduction. The confined enzyme cascade can be extended to include E4 etc. The principle can be divided into two steps.

In the first step (Figure 1A), the substrate for E2 is added to the cell solution (with NADP(H) already present) to initiate the coupled FNR−E2 reaction: NADPH produced by E2 is immediately re‐oxidized back to NADP^+^ by FNR resulting in a current, directly proportional to the turnover rate of E2, which quickly reaches a steady state level. In the second step, the E3‐catalysed reduction reaction is initiated by injecting its substrate (Figure 1B): E3 now competes with FNR for the NADPH supplied by E2, providing an alternative *non‐electrochemical* pathway for NADP^+^ recycling. The *counter current*, which is directly proportional to the E3 turnover rate, is measured as an attenuation of the oxidation current treated in isolation. Note that the combined action of E2 and E3 is an example of a hydrogen‐borrowing reaction^[27]^ that is self‐sustaining in homogeneous solution; here, however, it acts to provide an internal counter‐circuit once E3 activity is engaged.

**Scheme 1:**
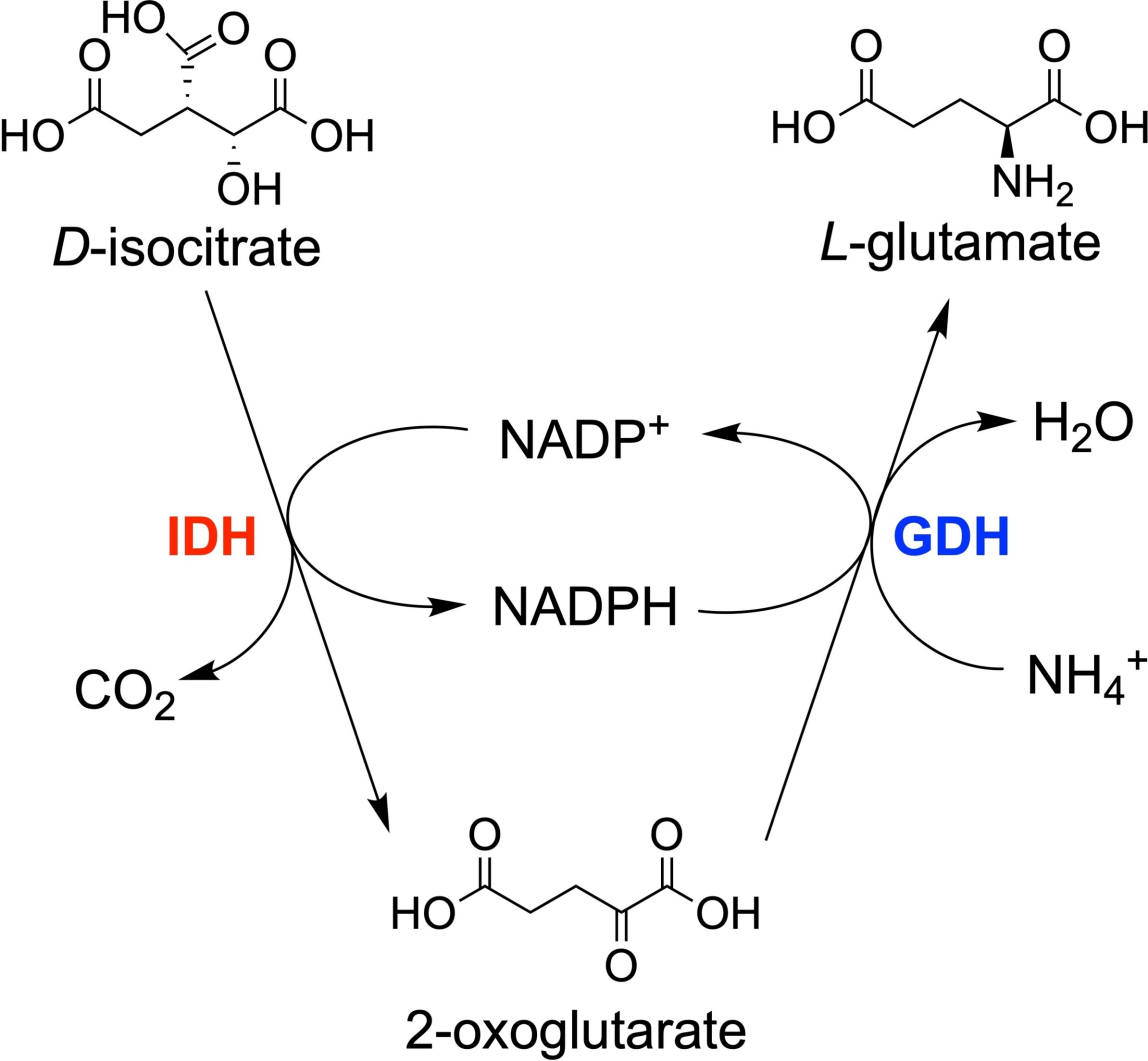
Without any additional redox agents, hydrogen‐borrowing between IDH (E2) and GDH (E3) results in catalysis with zero net consumption of NADP(H).

We envisaged that a nanoconfined hydrogen‐borrowing circuit, comprising an isocitrate dehydrogenase as E2 and a glutamate dehydrogenase as E3, could be established (Scheme 1). Isocitrate dehydrogenase I (IDH1) catalyzes the NADP^+^‐dependent oxidation of isocitrate to 2‐oxoglutarate (2OG) and CO_2_ (formal potential, −0.41 V at pH 8).^[28]^ Glutamate dehydrogenase (GDH) catalyzes the NADPH‐dependent reductive amination of 2‐oxoglutarate (2OG) to form *L*‐glutamate (formal potential, −0.196 V at pH 8, Figure S1). The activities of IDH1[^3^, ^22^, ^28^] and GDH[^6^, ^12^] have each been studied using the e‐Leaf. The strategic choice of these E2 and E3 enzymes offered a further level of control in that 2OG is both a product of IDH1 and a substrate of GDH; thus, GDH activity cannot exceed that of IDH1 (Scheme 1).

### Demonstrations

**Figure 2:**
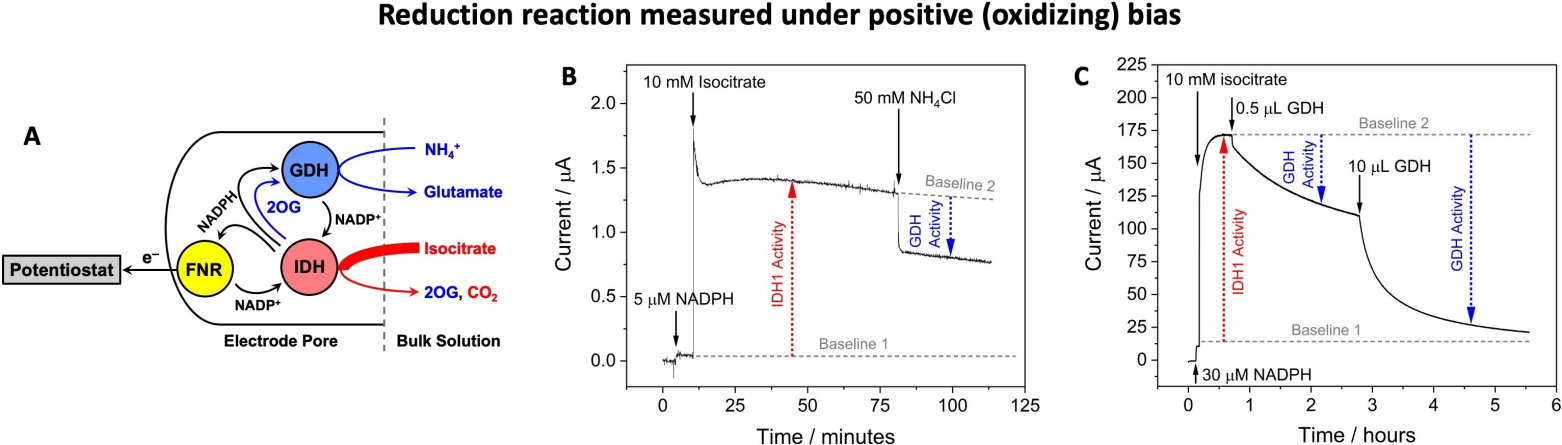
Non‐linear enzyme cascade enabling a reduction reaction to be monitored under a positive (oxidizing) bias. (**A**) Cascade map showing how GDH offers an alternative (non‐electrochemical) NADP^+^ recycling pathway. (**B**) All 3 enzymes were co‐loaded prior to start of the experiment. IDH1 oxidation was initiated by *DL*‐isocitrate addition. GDH reduction was then initiated by NH_4_^+^ addition causing GDH to compete with FNR for NADPH resulting in measurement of a counter current proportional to GDH activity. (**C**) Experiment with a large electrode (4 cm^2^) in which FNR and IDH1 were co‐loaded prior to starting the experiment. GDH was then injected into the bulk solution – the counter current (due to GDH reductive amination) increased slowly over time as GDH entered the electrode nanopores. The initial small sharp drop in the oxidation current is attributable to GDH‐catalyzed consumption of NADPH in bulk solution (NADPH diffusing from bulk solution produces a small background oxidation current) by reaction with 2OG produced by IDH1 (the 2OG solution concentration was ~0.37 mM at this point, based on the charge passed). See **Supporting Information** for detailed experimental conditions and repeats of the experiments (Figure S3).

**Figure 3:**
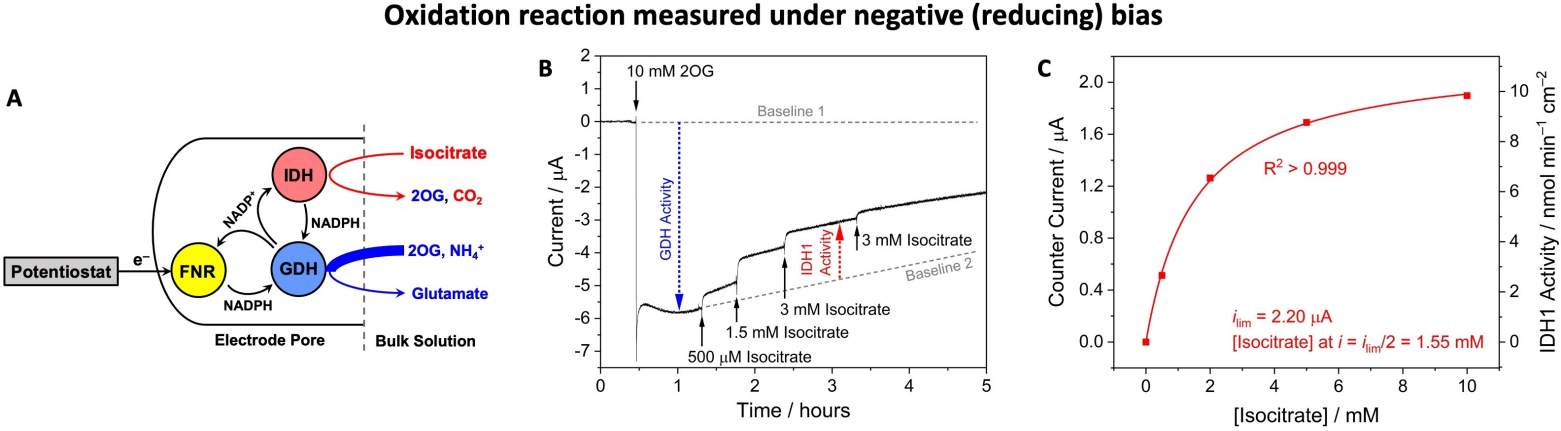
Non‐linear enzyme cascade enabling an oxidation reaction to be monitored under a negative (reducing) bias. (**A**) Cascade map showing how IDH1 offers an alternative (non‐electrochemical) NADPH recycling pathway. (**B**) The 3 enzymes were co‐loaded into the electrode. GDH activity was initiated by addition of 2OG. IDH1 activity was then initiated via additions of increasing concentrations of *DL*‐isocitrate yielding a counter current proportional to IDH1 activity as IDH1 competed with FNR for NADP^+^. (**C**) Plot of counter current vs [isocitrate] with data fit to Eq. (1). See **Supporting Information** for detailed experimental conditions.

**Figure 4:**
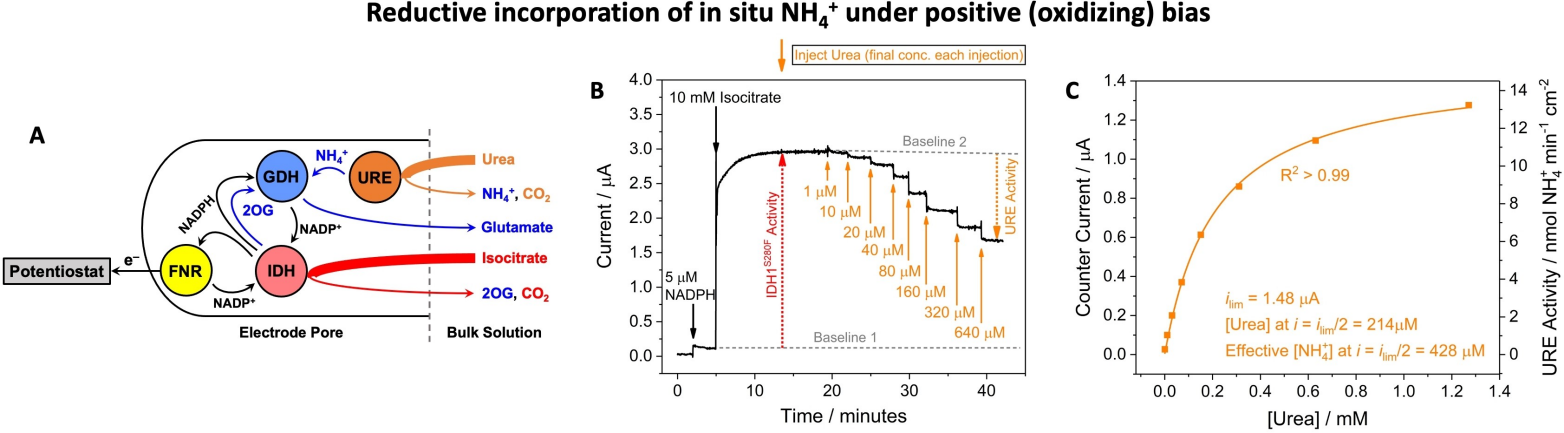
Extended 4‐enzyme cascade to detect hydrolysis of urea via a reductive amination reaction under a positive (oxidizing) bias. (**A**) Cascade map showing how urease activity is measured as counter current by activating the GDH‐dependent non‐electrochemical NADP^+^ recycling pathway. (**B**) All 4 enzymes were co‐loaded into the electrode before the start of the experiment. IDH1^S280F^ activity was initiated by injecting *DL*‐isocitrate. Urea was then titrated into the reaction solution and urease activity (i.e., urea hydrolysis generating in situ ammonia) was measured as counter current via the reductive amination of 2OG to glutamate catalyzed by GDH as GDH competed with FNR for NADPH substrate. (**C**) Plot of counter current vs [urea] with data fit to Eq. (1). See **Supporting Information** for detailed experimental conditions and a repeat of the experiment (Figure S7).

The consequences of introducing a hydrogen‐borrowing circuit to modulate a tightly‐channeled nicotinamide current are summarized in Figures 2, 3, 4. Unless otherwise stated, the following general conditions applied: a rotating disc pyrolytic graphite edge (PGE) electrode (0.03–0.06 cm^2^) on which was deposited a porous ITO layer (see Supporting Information); electrode rotation rate 1000 rpm; cell solution volume, 4 mL; high buffer concentration comprised of MES, HEPES, TAPS or combination thereof with 10 mM MgCl_2_ (required by IDH1); temperature 25 °C. All electrode potentials are referenced to the standard hydrogen electrode (SHE) scale. Enzymes were loaded on the electrode by dropcasting concentrated mixtures in empirically optimized molar ratios reflecting the varying inherent activities of each enzyme. The electrode was thoroughly rinsed before introducing it to the cell solution; NADP(H) and substrates were already present or were subsequently added as indicated by solid arrows in Figures 2, 3, 4.

#### Reduction Under a Positive (oxidizing) Bias

Figure 2 shows how the oxidation current, resulting from FNR‐catalyzed electrochemical oxidation of the NADPH that is continuously regenerated by IDH1 via isocitrate oxidation, is attenuated by a competing process: reductive amination of 2OG catalyzed by GDH consumes and recycles local NADPH before it can be recycled electrocatalytically by FNR. The cascade map is shown in Figure 2A. Figure 2B shows how the current responds to a sequence of additions after the electrode has been loaded with FNR, IDH1 and GDH in a 2 : 1 : 1 molar ratio (see Supporting Information for details). The electrode was placed into a solution buffered at pH=8.0, and an oxidizing potential was applied (+0.11 V vs SHE). Injection of NADPH (5 μM) into the cell solution produced a very small oxidation current due to its oxidation by FNR at the electrode. Subsequent introduction of *DL*‐isocitrate (10 mM) then resulted in a large oxidation current. The reaction was allowed to continue for >1 hour to establish stability. Injection of the GDH substrate NH_4_^+^ (as NH_4_Cl) into the cell solution resulted in a rapid and substantial decrease in oxidation current (~40 % of the IDH1‐alone rate), implying that the GDH competes with FNR for the NADPH produced by IDH1. Control experiments (without GDH) showed no change in oxidation current after NH_4_Cl addition (Figure S2).

To investigate whether the decrease in IDH1 oxidation current reflects the counter current from GDH activity, an experiment was performed using a large (4 cm^2^) electrode into which only FNR and IDH1 were loaded (2 : 1, respectively) (Figure 2C). After rinsing, the electrode was placed in the cell solution, an oxidizing potential (+0.21 V) was applied, and NADPH (30 μM) was added to the cell solution. Isocitrate was then introduced, resulting in a large oxidation current; GDH was then injected into the cell solution (final concentration: 65 nM). An initial small, sharp drop in oxidation current (see legend) was followed by a much greater decrease over several hours, due not to consumption of reactants, but to an increase in the rate of the competing reaction as GDH enters and becomes concentrated in the electrode pores.^[7]^ After approximately 2 hours, when the current was still decreasing slowly, a larger quantity of GDH was injected into the cell solution (final concentration: 1.3 μM), resulting in an accelerated decrease in the net current.

#### Oxidation Under a Negative (reducing) Bias

Figure 3 demonstrates the reverse situation, i.e. measurement of the catalytic oxidation of isocitrate at a negatively poised (reducing) electrode, but with anaerobic conditions imposed by an atmosphere of N_2_. After co‐loading FNR, IDH1, and GDH at a (respective) 2 : 1 : 1 molar ratio, the electrode was rinsed and placed in the cell solution (4 mL) containing 0.1 M NH_4_Cl and 10 μM NADPH. A reducing potential was applied (−0.47 V) and 2OG (10 mM) was added, resulting in a reduction current as FNR recycled NADPH for the catalytic conversion of 2OG to *L*‐glutamate by GDH (Figure 3B). Once the FNR‐GDH reduction rate had reached a steady‐state, the IDH1‐catalyzed isocitrate oxidation reaction was initiated by stepwise additions of isocitrate, each of which resulted in rapid but diminishing attenuations of the current, consistent with the turnover rate of the competing enzyme (IDH1) approaching a maximum value. Measurements of counter current (*i*), which represents IDH1 activity, at different concentrations of isocitrate are shown in Figure 3C. The plot showed a good fit to the empirical hyperbolic equation (1) where *i*_lim_ is the limiting current, [S] is the cell concentration of *DL*‐isocitrate, and *x* is the substrate concentration required to achieve *i*=*i*_lim_/2.

(1) $$i=\frac{i_{lim}\left[S\right]}{x+\left[S\right]}$$

Note that although equation (1) resembles the Michaelis–Menten equation, the conditions for using the latter in the normal way are not met in the e‐Leaf situation (see below).

#### Extending to Incorporate Localized Ammonia Production

Figure 4 describes extending the cascade to include the Ni‐containing hydrolase, urease, to generate in situ ammonia for use by GDH. The system was set up as for Figure 2B except that: (i) a two‐fold more active IDH1 variant, IDH1^S280F^,^[29]^ was used in place of wildtype IDH1 to decrease (by half) the amount loaded, and (ii) the amount of GDH loaded was doubled to compensate for the fact that all of its substrates would now be supplied in situ by other enzymes (Figure 4A); the molar loading ratio used was FNR/IDH1^S280F^/GDH/URE=4/1/4/0.2. From the corresponding “non‐turnover” CV measured before catalytic action was initiated, the FNR coverage was estimated to be 334 pmoles/cm^2^ (Figure S4) implying an average FNR concentration of 1.1 mM spread throughout a 6 μm depth at 50 % pore volume. Based on a report explaining how the concentration of IDH1 could also be estimated for given loading ratios,^[3]^ it is likely that IDH1 and GDH also have pore concentrations in the millimolar region, with a lower value applying for urease, compensated for by the fact that it is a highly active enzyme.^[30]^ The oxidation reaction was initiated by adding 10 mM *DL*‐isocitrate and was allowed to reach a steady state over the course of 20 min. Figure 4B shows the changes in current recorded as urea was titrated into the solution. Each addition resulted in an immediate decrease in oxidation current as GDH diverted some of the steady‐state flux of NADPH away from FNR – note that the 2OG also arises from IDH1 activity. The individual response times are displayed in Figure S5. The plot of counter current (representing urease activity) against urea concentration (Figure 4C **)** gave a good fit to the simple hyperbolic equation (1) (R^2^ > 0.99) yielding *x*=214 μM, A control (Figure S6) without GDH in the electrode showed no substantial changes in the oxidation current following additions of urea.

The hyperbolic dependence of counter current on the bulk concentration of a reactant shows that measurements can be calibrated for specific sets of conditions. However, the quantity *x* is not expected to relate closely to the Michaelis constant for the enzyme responsible, as conditions are far removed from those normally applied for Michaelis–Menten kinetics.[^31^, ^32^, ^33^] Aside from the uncertain consequences of enzyme crowding,[^2^, ^4^] a high enzyme concentration in the electrode would mean that locally there may be more enzyme available than substrate molecules to process. This situation is exacerbated if the enzyme is very active as it will result in rapid depletion of local substrate. Accordingly, a repeat experiment of Figure 4B in which the counter current was approximately 2‐fold greater (higher enzyme activity) gave proportionately higher values of x (Figure S7). Such an excess of enzyme over substrate likely often applies in vivo.^[34]^ For IDH1, reported *K*_M_ values for isocitrate are <0.1 mM and turnover frequencies exceed 50 s^−1^.[^29^, ^35^, ^36^] Any limitation from diffusion of reactants through the pores would be relaxed for less‐active enzymes or with reagents that are not catalytically consumed, i.e. inhibitors, or NADP(H) which is locally recycled: but with a high turnover rate, the demand for isocitrate imposed by the high density of IDH1 enzymes in the pores would far exceed its supply. For urease, the hyperbolic dependence on urea concentration is, likewise, very useful for calibration; however, the role of urease in this case is to generate in situ NH_4_^+^ to supply GDH, and attaching any more detailed kinetic significance is unjustified at this stage.

## Conclusions

Once nanoconfined and highly localized within the ITO electrode material, nicotinamide cofactors serve as discrete, two‐way current‐carriers, bringing to biocatalysis capabilities that are normally associated with electronic circuits – in this case a rapid response time following a remote stimulus and measurement of a process opposed to the potential bias. The hydrolytic activity of urease, which is linked to an enzyme that competes with FNR for local NADPH, is immediately registered – the response time being seconds or less even at urea concentrations <10 μM (Figure S5). Detecting and monitoring biocatalytic reduction reactions in electrochemical sensors usually requires anaerobic conditions because the electrode or mediators also reduce O_2_. This limitation is now overcome by incorporating a hydrogen‐borrowing circuit which acts as a pivot, distributing NADP(H) between two competing recycling pathways and allowing the reduction reaction to be observed as an attenuating counter current, even under aerobic conditions.

**Scheme 2:**
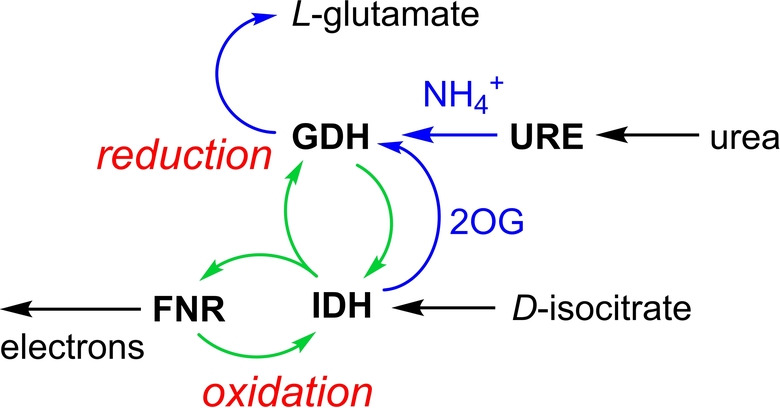
Role of the nanoconfined hydrogen‐borrowing circuit in enabling the action of a reductive aminase to be observed electrochemically under oxidizing conditions. FNR=E1, IDH=E2, GDH=E3, URE=E4. External reactants are *D*‐isocitrate and urea. Green arrows represent the internal NADP(H) circuit, blue arrows represent pathways of internal intermediates (CO_2_ and H_2_O omitted).

The flow of reactions within the nanoconfined hydrogen‐borrowing enzyme cascade is summarized in Scheme 2 in a way that distinguishes between the different internal components: oxidation and reduction (red), NADP(H) recycling (electrochemical and hydrogen‐borrowing) circuits (green), and the reduction reaction that is detected (blue) at an oxidizing electrode.

That certain individual electron‐transport enzymes can behave as electronic components has long been known; indeed, succinate dehydrogenase displays characteristics of a tunnel diode.[^37^, ^38^] The e‐Leaf, however, goes much further by operating at the level of entire cascade systems requiring the collective and connective properties of multiple enzymes. Enzymes of all major classes and size (GDH and urease are large oligomeric enzymes) may be incorporated, offering new possibilities for studying their properties via rapid, interactive dialogue. The hydrogen‐borrowing system reported here is convenient because it is based on commercially available enzymes, but many more opportunities for “cascadetronics” exist.^[39]^ Indeed, the typical high activities and selectivities of enzymes, coupled with the interactive energization and nanoconfinement of cascades that integrate them, facilitates their potential applications as logic gates – various options for which have been proposed by Katz and others.[^15^, ^40^, ^41^]

The ease and speed of investigating the initiating reaction of the extended 4‐enzyme cascade, i.e. detecting the rapid in situ generation of ammonia, may itself have further significance. Deaminases are widespread in biology as catalysts for both oxidative and non‐oxidative transformations of amino acids and the components and precursors of nucleic acids.[^42^, ^43^, ^44^] The ability to manipulate such enzymes via nanoconfinement – resulting in a rapid and informative electrochemical response – opens up new avenues for their investigation and exploitation.

Within the e‐Leaf, the NADP(H) currents and counter currents, representing both oxidative *and* reductive enzyme pathways in complex cascades, can be quantified within single experiments that demonstrate and highlight the speed at which collective enzyme systems respond to a small stimulus. It is important to stress that a sequence of reactions involving several enzymes can be driven in either direction with a rapid switching capability that (aided by the absence of electron mediators) is virtually devoid of inertia. The results open the way to investigating the effects of modulation, not only on the efficiency of the overall nanoconfined cascade, but (with appropriate controls) on sets of reactions therein. In addition to classical small‐molecule modulators (such as active‐site binding inhibitors), *inter alia* it will be of interest to study the effects of enzyme enhancers, uncompetitive inhibitors, crowding reagents, and apparently pleiotropically acting redox active metabolites (e.g. ascorbate, flavonoids).

Finally, the methodology has the potential to model how NAD(P)(H) status is regulated in different localized environments within cells. That nanoconfined NADP(H) is such an effective current carrier is relevant to the questions of if and how cofactor fluxes are localized in cell metabolism.[^45^, ^46^, ^47^, ^48^, ^49^, ^50^, ^51^]

## Supporting Information

The authors have cited additional references within the Supporting Information.[^52^, ^53^, ^54^]

## Conflict of Interests

The authors declare no conflict of interest.

1

## Supporting information

As a service to our authors and readers, this journal provides supporting information supplied by the authors. Such materials are peer reviewed and may be re‐organized for online delivery, but are not copy‐edited or typeset. Technical support issues arising from supporting information (other than missing files) should be addressed to the authors.

Supporting Information

## Data Availability

The data that support the findings of this study are available in the supplementary material of this article.
