# Supplementary material for: Building Localized NADP(H) Recycling Circuits to Advance Enzyme Cascadetronics
Source: Angew Chem Int Ed Engl. 2025 Feb 11;64(10):e202414176. doi: 10.1002/anie.202414176 (PMC11878340; doi:10.1002/anie.202414176)
Supplement: Supplementary file 1 — Supporting Information [file ANIE-64-e202414176-s001.pdf]

Angewandte Chemie
Eine Zeitschrift der Gesellschaft Deutscher Chemiker

Supporting Information

Building Localized NADP(H) Recycling Circuits to Advance Enzyme Cascadetronics

R. A. Herold, C. J. Schofield, F. A. Armstrong**

Supporting Information

Building Localized NADP(H) Recycling Circuits to Advance Enzyme Cascadetronics

Ryan A. Herold,*[a]† Christopher J. Schofield,[a,b] and Fraser A. Armstrong*[a]

[a]Department of Chemistry, University of Oxford, Oxford OX1 3QR, United Kingdom.

[b]Department of Chemistry and the Ineos Oxford Institute for Antimicrobial Research, University of Oxford, Oxford OX1 3QY, United Kingdom.

†Current Address: Department of Chemistry and Biochemistry, University of California, San Diego, La Jolla, CA 92093, USA.

Materials and Methods

Chemicals

$NADP^+$ (monosodium salt, 98%, Melford), NADPH (tetrasodium salt, 93%, Melford), acetone (ACS Reagent), indium tin oxide (ITO) powder (< 50 nm particle size, Sigma-Aldrich), (HEPES) (free acid, Melford), [2-(N-morpholino)-ethanesulfonic acid] (MES) (monohydrate, Melford), [tris(hydroxymethyl)methylamino]-propanesulfonic acid (TAPS) (99%, Melford), [2-(N-cyclohexylamino)ethanesulfonic acid] (CHES) (>99%, Melford), [N-(2-hydroxyethyl) piperazine N'-(2-ethanesulfonic acid)], 2-oxoglutarate (disodium salt dihydrate, ≥98%, Sigma-Aldrich), *DL*-isocitric acid (trisodium salt hydrate, ≥93%, Sigma-Aldrich), ammonium chloride (99.5% minimum, Thermo Scientific Chemicals), urea (ACS, 99.0-100.5%, Thermo Scientific Chemicals), $MgCl_2$ (anhydrous, Melford), iodine (resublimed crystals, 99.9985% (metals basis), puratronic, Alfa Aesar), isopropyl β-D-1-thiogalactopyranoside (IPTG) (Alfa Aesar). Aqueous solutions were made using ultrapure water (≥18.2 MΩ cm, Milli-Q).

Enzyme Expression and Purification

Human isocitrate dehydrogenase 1 (wildtype and the S280F variant) and ferredoxin $NADP^+$-reductase (FNR) from *Chlamydomonas reinhardtii* were recombinantly expressed and purified as previously described.[1,2] Glutamate dehydrogenase from *E. coli* was expressed in *E. coli* and purified following the procedure described by Cheng et al.[3] Urease from *Canavalia ensiformis* (jack bean) was purchased from Sigma-Aldrich (product no. 666133) as a lyophilized powder.

Electrodes

A thin mesoporous layer (3–6 μm) of indium tin oxide (ITO) was formed on either pyrolytic graphite edge (PGE) rotating disc electrodes or Ti foil by electrophoretic deposition of ITO nanoparticles (<50 nm, Sigma-Aldrich) as previously described.[4] In-house rotating disc electrodes (PGE) were prepared as reported.[5] ITO-modified electrodes were rinsed with water before loading with enzyme.

Enzyme Loading

Enzymes were loaded into the nanoporous ITO electrodes as previously reported.[1,6] Briefly, a concentrated mixed enzyme solution (4–8 µL total) was pipetted onto the electrode surface and the treated material was incubate for 30–45 minutes at room temperature. During the enzyme loading process, each electrode was covered with a 1.5 ml Eppendorf tube to minimize evaporation of the enzyme solution. Electrodes were rinsed thoroughly with buffer prior to use to ensure that any non-adsorbed enzyme was removed. In each experiment, 0.85 nmoles of dimeric IDH1 (wildtype or the S280F variant) was used; the amounts of the other enzymes loaded were adjusted to achieve the desired ratios (see **Experimental Details Corresponding to Figures 2–4** below). The same amount of IDH1 enzyme was applied to the larger 4 cm^2 Ti foil electrode for the experiment shown in **Figure 2C** (main text) as was used for the smaller rotating disc electrodes. The concentration of IDH1 (wildtype and S280F) was calculated based on the active dimeric form of the enzyme which is reported to display half-site reactivity.[7] All other enzyme concentrations were calculated based on the number of monomer equivalents.

General Experimental Conditions

Electrochemical experiments were performed as reported[6] using an Autolab PGSTAT 10 potentiostat and Nova software. Experiments requiring the application of a reducing electrode potential were performed in an anaerobic glove box (Braun Technologies) containing a nitrogen atmosphere (O_2 < 1 ppm). A two-chamber glass electrochemical cell was used for the analytical-scale experiments: the small ITO/PGE rotating disc electrode (working electrode) and platinum counter electrode were present in the same chamber; the reference electrode was housed in a separate chamber containing 0.1 M NaCl.[1,5,6] The experiment described in **Figure 2C** utilized a three-chamber electrochemical cell in which each electrode was housed in a separate chamber.[8] The temperature of the working electrode chamber was maintained using a water jacket connected to a circulating water bath. Electrode potentials were measured against a saturated calomel electrode (SCE) and converted to SHE using a temperature-dependent conversion table.[1]

Experimental Details Corresponding to Figures 2–4

Figure 2B: all 3 enzymes were co-loaded prior to the start of the experiment; the GDH reaction was initiated by injection of aliquots of 1.5 M NH_4Cl solution (final solution concentration, 50 mM). Conditions: (FNR+IDH1+GDH)@ITO/PGE electrode, electrode area 0.03 cm^2, electrode rotation rate 1000 rpm, 25 °C, pH = 8 (50 mM each: MES, TAPS), 10 mM $MgCl_2$, 5 µM NADPH, E = +0.11 V vs SHE, 3 mL volume, enzyme loading ratios (molar): FNR/IDH1/GDH; 2/1/1.

Figure 2C: FNR and IDH1 were co-loaded prior to the start of the experiment; the GDH reaction was initiated by injecting GDH enzyme into the reaction solution from which it was slowly taken up by the electrode. Conditions: (FNR+IDH1)@ITO/Ti foil electrode, electrode area 4 cm^2, 25 °C, pH = 7.5 (100 mM HEPES), 10 mM $MgCl_2$, 100 mM NH_4Cl, 50 µM NADPH, E = 0.21 V vs SHE, 4 mL volume, enzyme loading ratios (molar): FNR/IDH1; 2/1. The solution was mixed using a stir bar. The concentration of the GDH solution added was 0.52 mM.

Figure 3B: all 3 enzymes were co-loaded in the electrode prior to the start of the experiment; the IDH1 reaction was initiated by injecting increasing concentrations of *DL*-isocitrate. Conditions: (FNR+IDH1+GDH)@ITO/PGE electrode, electrode area 0.06 cm^2, electrode rotation rate 1000 rpm, 25 °C, pH = 7.5 (50 mM HEPES), 10 mM $MgCl_2$, 10 µM NADPH, E = –0.47 V vs SHE, O_2 < 1 ppm, 4 mL volume, enzyme loading ratios (molar): FNR/IDH1/GDH; 2/1/1.

Figure 4B: all 4 enzymes were co-loaded prior to the start of the experiment; urease activity was measured as counter current (via the reductive amination of 2OG catalyzed by GDH which competed with FNR for NADPH) as urea was titrated into the bulk solution. Conditions: (FNR+$IDH1_{S280F}$+GDH+URE)@ITO/PGE electrode, electrode area 0.03 cm^2, electrode rotation rate 1000 rpm, 25 °C, pH = 8 (200 mM TAPS), 10 mM $MgCl_2$, 5 µM NADPH, E = 0.041 V vs SHE, 4 mL volume, enzyme loading ratios (molar): FNR/$IDH1_{S280F}$/GDH/URE; 4/1/4/0.2.

Supporting Results (Figures)

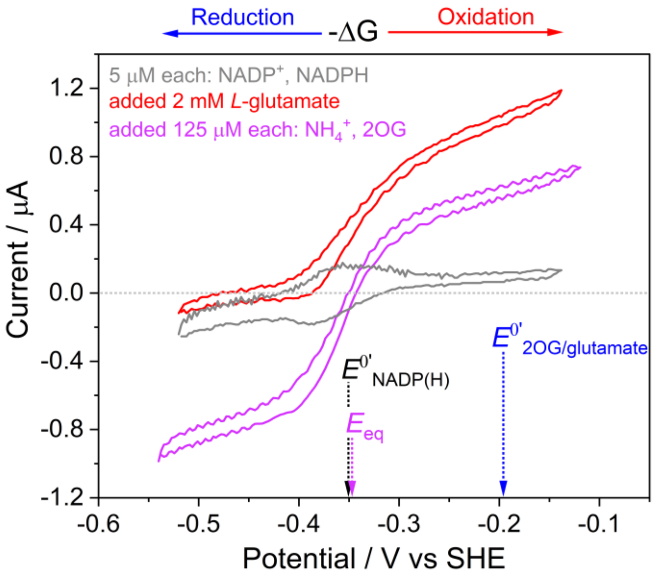

Figure S1. 2OG-NH_4^+/glutamate formal reduction potential estimated by finding a combination of components that equalize the current in each direction (oxidation and reduction) and applying the Nernst equation. The results show that the formal potential of the 2OG-NH_4^+/glutamate couple at pH = 8 is approximately –0.196 V; literature value at pH = 7 is –0.126 V[9], equivalent to –0.186 at pH 8 assuming a 2e$^-$, 2H$^+$ reaction. Conditions: (FNR+GDH)@ITO/PGE electrode, electrode area 0.03 cm^2, electrode rotation rate 1000 rpm, scan rate 2 mV s^{-1}, 25 °C, pH = 8 (200 mM TAPS), 5 µM each: NADP$^+$ and NADPH, O_2 < 1 ppm, 4 mL volume, enzyme loading ratios (molar): FNR/GDH; 1/1.

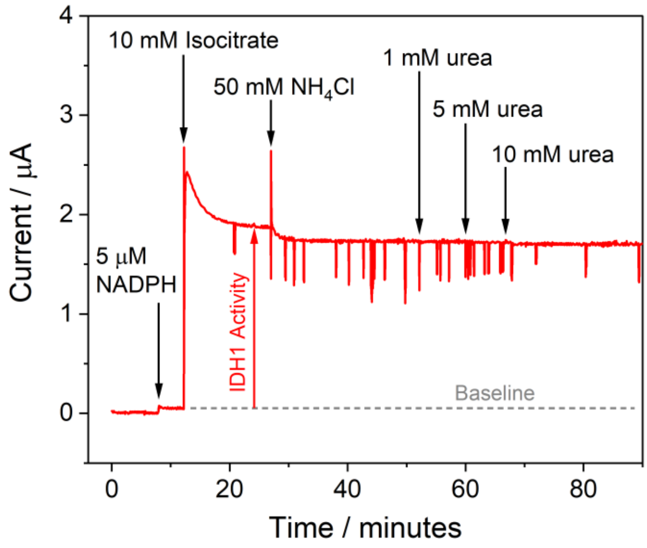

Figure S2. Chronoamperometry experiment showing that additions of NH_4^+ and urea do not affect FNR or IDH1 activity when GDH is not present in the electrode. Conditions: (FNR+IDH1)@ITO/PGE electrode, electrode area 0.03 cm^2, electrode rotation rate 1000 rpm, 25 °C, pH = 8 (200 mM TAPS), 10 mM $MgCl_2$, 5 µM NADPH, E = 0.041 V vs SHE, 4 mL volume, enzyme loading ratios (molar): FNR/IDH1; 2/1.

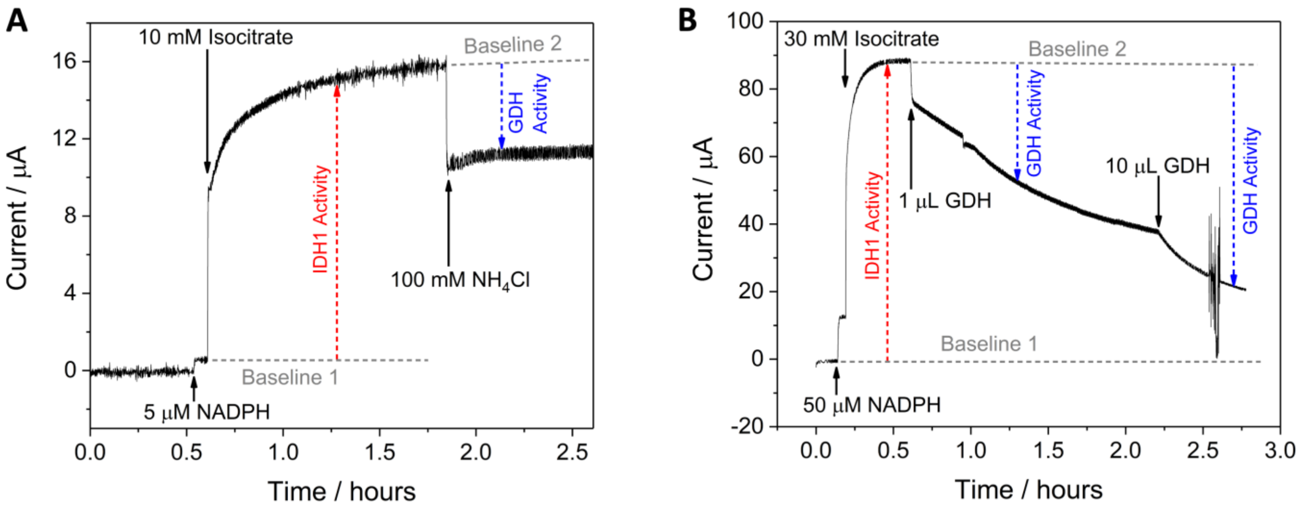

Figure S3. Repeats of the experiments shown in **Figure 2** in the main text. (A) Repeat of experiment shown in **Figure 2B**: all 3 enzymes were co-loaded prior to start of the experiment, IDH1 oxidation was initiated by *DL*-isocitrate addition, and GDH reduction was initiated by NH_4^+ addition. (B) Repeat of experiment shown in **Figure 2C**: FNR and IDH1 were co-loaded prior to starting the experiment. GDH was injected into the bulk solution, and the counter current (due to GDH reductive amination competing with FNR for NADPH) increased slowly over time as GDH entered the electrode nanopores. The initial sharp drop in oxidation current was due to GDH-catalyzed consumption of NADPH in bulk solution (NADPH diffusing from bulk solution produces a small background oxidation current) by reaction with 2OG produced by IDH1. Conditions: 25 °C, pH = 8 (50 mM each: MES, TAPS), 10 mM $MgCl_2$, 3 mL volume, solution mixed with stir bar, electrode area 4 cm^2. (A) (FNR+IDH1+GDH)@Ti foil electrode, enzyme loading ratios (molar): FNR/IDH1/GDH; 1/1/4.5, E = +0.04 V vs SHE. (B) Conditions: (FNR+IDH1)@ITO/Ti foil electrode, FNR/IDH1; 1/1, E = +0.2 V vs SHE. The concentration of the GDH solution added was 0.52 mM.

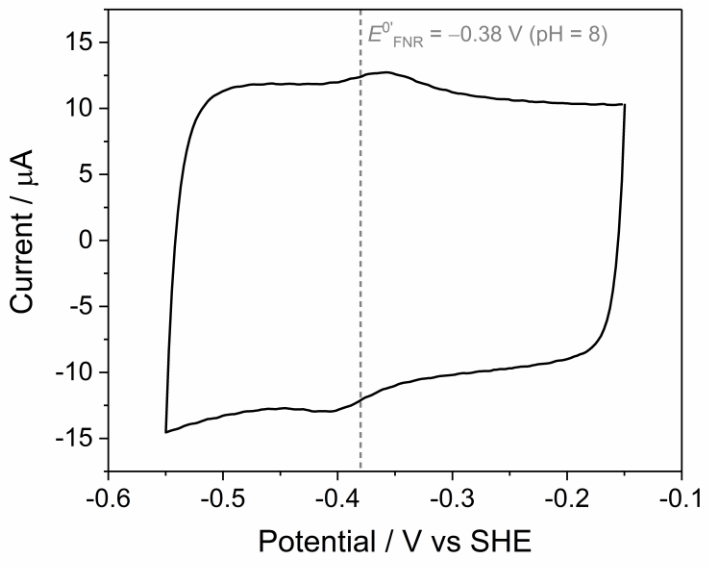

Figure S4. Cyclic voltammogram showing the FNR non-turnover peaks (reduction and re-oxidation of the FAD prosthetic group via direct electron exchange with the working electrode)[8,10,11] for the experiment shown in **Figure 2H** in the main text. The FNR coverage (334 pmoles cm^{-2}) was determined by integrating and averaging the non-turnover peaks as has been previously described.[1]

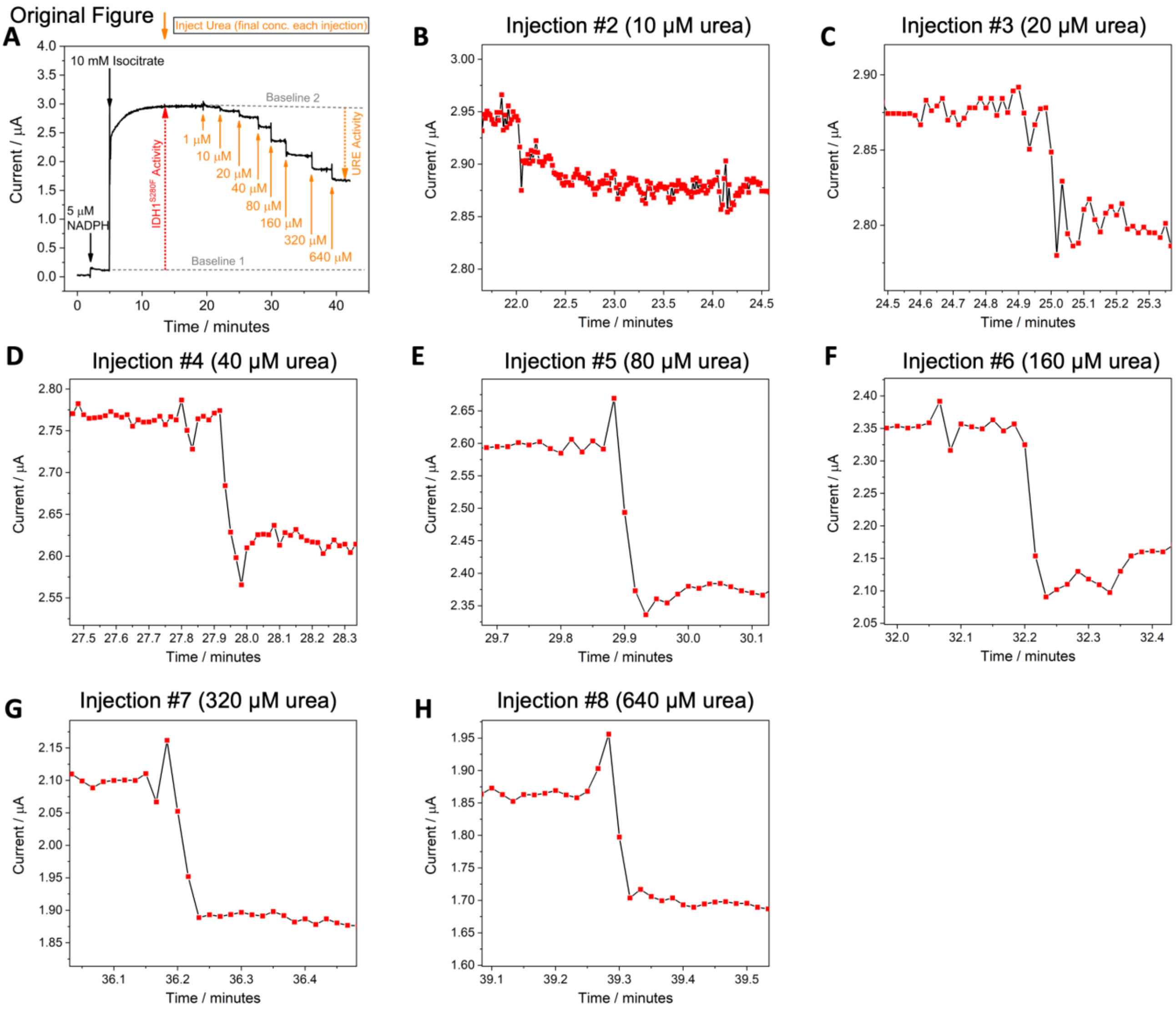

Figure S5. Response times following each urea injection after the first 1 µM addition for the experiment shown in **Figure 4B** in the main text (reshown here in panel A).

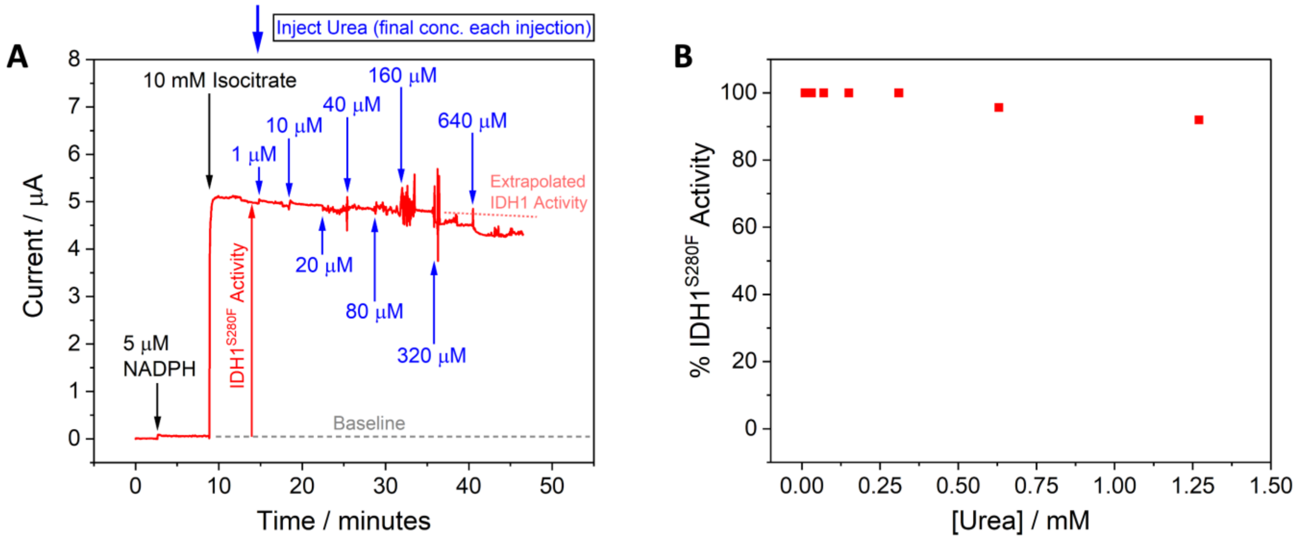

Figure S6. Control experiment showing that additions of urea do not significantly affect FNR or IDH1^{S280F} activity when GDH is not present in the electrode but urease (URE) *is* present. (A) Chronoamperogram showing additions of increasing concentrations of urea (each injection indicates the added cell concentration of urea for each addition (total urea concentration is thus the injected value plus all previous additions). (B) Percentage of remaining IDH1^{S280F} activity following each injection of urea for the experiment shown in panel A. Conditions: (FNR+IDH1^{S280F}+URE)@ITO/PGE electrode, electrode area 0.03 cm^2, electrode rotation rate 1000 rpm, 25 °C, pH = 8 (200 mM TAPS), 10 mM $MgCl_2$, 5 μM NADPH, *E* = 0.041 V vs SHE, 4 mL volume, enzyme loading ratios (molar): FNR/IDH1^{S280F}/URE; 4/1/0.1.

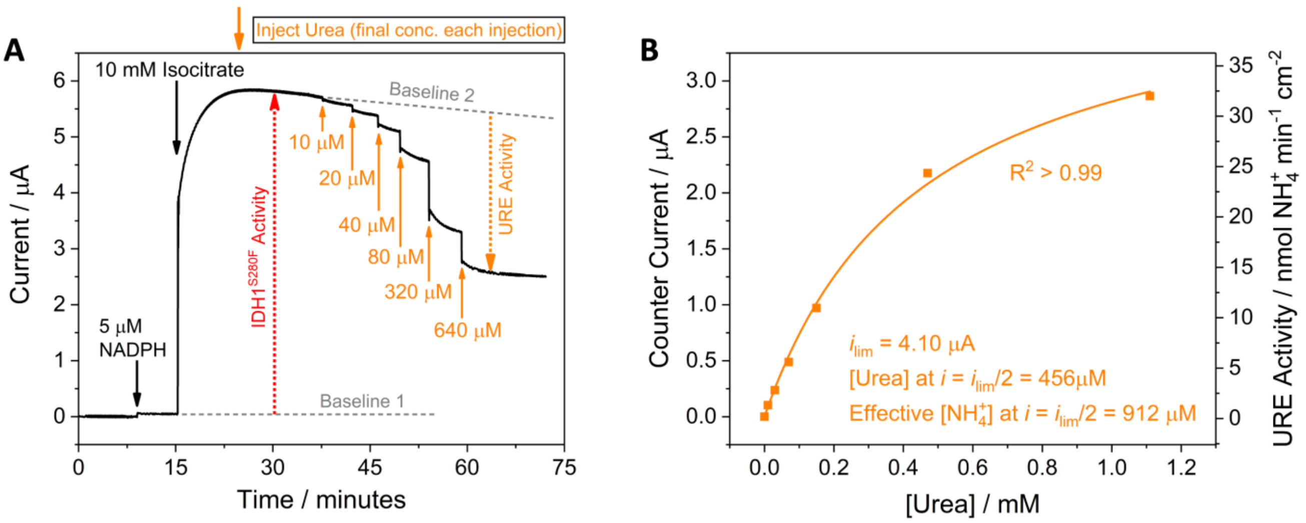

Figure S7. Repeat of the experiment shown in **Figure 4** in the main text. (A) All 4 enzymes were co-loaded into the electrode before starting the experiment. IDH1^{S280F} activity was initiated by injecting *DL*-isocitrate. Urea was titrated into the solution, and urease activity was measured as counter current via the reductive amination of 2OG to glutamate catalyzed by GDH as GDH competed with FNR for NADPH substrate. (B) Plot of counter current vs [urea] fit by Eq. (1). Conditions: (FNR+IDH1$_{S280F}$+GDH+URE)@ITO/PGE electrode, electrode area 0.03 cm^2, electrode rotation rate 1000 rpm, 25 °C, pH = 8 (200 mM TAPS), 10 mM $MgCl_2$, 5 μM NADPH, *E* = 0.041 V vs SHE, 4 mL volume, enzyme loading ratios (molar): FNR/IDH1$_{S280F}$/GDH/URE; 4/1/4/0.2.

Supporting References

[1] R. A. Herold, R. Reinbold, C. F. Megarity, M. I. Abboud, C. J. Schofield, F. A. Armstrong, *J. Phys. Chem. Lett.* **2021**, *12*, 6095–6101.

[2] R. Reinbold, I. C. Hvinden, P. Rabe, R. A. Herold, A. Finch, J. Wood, M. Morgan, M. Staudt, I. J. Clifton, F. A. Armstrong, J. S. O. McCullagh, J. Redmond, C. Bardella, M. I. Abboud, C. J. Schofield, *Nat. Commun.* **2022**, *13*, 4785.

[3] B. Cheng, L. Wan, F. A. Armstrong, *ChemElectroChem* **2020**, celc.202001166.

[4] R. A. Herold, C. J. Schofield, F. A. Armstrong, *Angew. Chemie Int. Ed.* **2023**, e202309149.

[5] R. M. Evans, F. A. Armstrong, in *Methods Mol. Biol.*, Humana Press Inc., **2014**, pp. 73–94.

[6] R. A. Herold, R. Reinbold, C. J. Schofield, F. A. Armstrong, *Proc. Natl. Acad. Sci.* **2023**, *120*, e2214123120.

[7] J. V. Roman, T. R. Melkonian, N. R. Silvaggi, G. R. Moran, *Biochemistry* **2019**, *58*, 5366–5380.

[8] B. Siritanaratkul, C. F. Megarity, T. G. Roberts, T. O. M. Samuels, M. Winkler, J. H. Warner, T. Happe, F. A. Armstrong, *Chem. Sci.* **2017**, *8*, 4579–4586.

[9] P. C. Engle, K. Dalziel, *Biochem. J.* **1967**, *105*, 691–695.

[10] C. F. Megarity, B. Siritanaratkul, R. A. Herold, G. Morello, F. A. Armstrong, *J. Chem. Phys.* **2020**, *153*, 225101.

[11] F. A. Armstrong, B. Cheng, R. A. Herold, C. F. Megarity, B. Siritanaratkul, *Chem. Rev.* **2023**, *123*, 5421–5458.
